# Forgetfulness in adult attention‐deficit/hyperactivity disorder masks transient epileptic amnesia: a case report

**DOI:** 10.1002/pcn5.70003

**Published:** 2024-08-22

**Authors:** Takashi Fukao, Masaki Fujiwara, Yuto Yamada, Shinji Sakamoto, Yosuke Matsumoto, Manabu Takaki

**Affiliations:** ^1^ Department of Neuropsychiatry Okayama University Hospital Okayama Japan; ^2^ Okayama University Hospital Gender Center Okayama Japan; ^3^ Department of Neuropsychiatry Okayama University Faculty of Medicine, Dentistry and Pharmaceutical Sciences Okayama Japan

**Keywords:** anti‐seizure medications, attention‐deficit/hyperactivity disorder, electroencephalography, transient epileptic amnesia

## Abstract

**Background:**

Inattention due to attention‐deficit/hyperactivity disorder (ADHD) can lead to forgetfulness. Transient epileptic amnesia (TEA) can cause forgetfulness, similar to ADHD. We report a patient with ADHD who developed TEA.

**Case Presentation:**

The patient was a 40‐year‐old woman with ADHD. She has been prone to forgetfulness since childhood. Two years before visiting our outpatient clinic, she had begun to occasionally forget events that had occurred several days earlier. However, she was largely unaware of the emergence of new amnestic symptoms. She had also begun to experience various other amnestic symptoms 2 months before she visited our clinic, which prompted her to visit our outpatient clinic. The combination of a detailed interview, electroencephalography (EEG) examination, and consideration of TEA enabled us to diagnose her with TEA and provide treatment accordingly. In our patient, daily forgetfulness due to ADHD delayed the recognition of new additional forgetfulness attributed to TEA.

**Conclusion:**

Psychiatrists need to consider TEA when patients with ADHD present with changes in or exacerbation of forgetfulness.

## Graphical abstract

We report a patient with ADHD who developed TEA. In our patient, daily forgetfulness due to ADHD delayed the recognition of new additional forgetfulness attributed to TEA. Psychiatrists need to consider TEA when patients with ADHD present with changes or exacerbation of forgetfulness.

## BACKGROUND

Inattention due to attention‐deficit/hyperactivity disorder (ADHD) can lead to forgetfulness, which includes losing belongings and forgetting schedules. Transient epileptic amnesia (TEA) is a syndrome characterized by recurrent attacks of amnesia^1^ and also causes forgetfulness, similar to ADHD. In addition to amnestic attacks, patients with TEA may experience anterograde amnesia because of accelerated long‐term forgetting (ALF) or episodic memory loss, which is caused by autobiographical amnesia (AA) during the interictal phase.^2^ TEA is a treatable condition that responds well to anti‐seizure medication (ASM).^3^ In this report, we describe an adult patient with ADHD who had undetected TEA that began 2 years prior. To the best of our knowledge, this is the first case report of ADHD complicated by TEA.

## CASE PRESENTATION

The patient was a 40‐year‐old woman who had no developmental delay during childhood. She has been prone to forgetfulness since childhood and often exhibited impulsive behavior. In adulthood, her impulsive behaviors subsided, and inattention became the predominant symptom. In daily life, she was aware of her forgetfulness, such as forgetting her children's schedules and to report at work. Two years before visiting our outpatient clinic, she had begun to occasionally forget events that had occurred several days earlier (e.g., shopping or dining out with family). She had also begun to experience various other amnestic symptoms 2 months before she visited our clinic, such as forgetting work procedures and family trips, which prompted her to visit our outpatient clinic.

**Figure 1:**
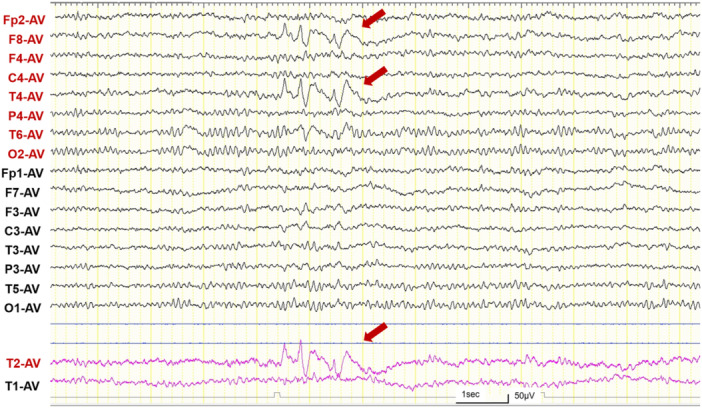
Electroencephalography (average reference) showing brief bursts of sharp‐and‐wave discharges over the right temporal region (red arrows) during an awake state. Yellow vertical lines represent 1‐s intervals. Sensitivity = 10 μV/mm; high‐frequency filter = 60 Hz; low‐frequency filter = 0.53 Hz.

The patient had no abnormal neurological findings or short‐term memory impairment. She scored 30 on the Mini‐Mental State Examination. Her full‐scale IQ as measured by the Wechsler Adult Intelligence Scale‐Fourth Edition was 94. Her scores on Conners' Adult ADHD Rating Scales exceeded the cutoff scores for all subscales. Although brain magnetic resonance imaging showed no abnormalities, electroencephalography (EEG) showed sharp‐and‐wave discharges over the right temporal region (Figure 1). We diagnosed her with ADHD according to the *Diagnostic and Statistical Manual of Mental Disorders*, Fifth Edition,^4^ based on clinical history. Her forgetfulness—which had been present since childhood—was considered a symptom of ADHD. The characteristic amnestic symptoms that the patient had been experiencing for the past 2 years were considered symptoms of ALF and AA, which occur during the interictal phase of TEA. Therefore, we started treatment with 50 mg of lacosamide, although she did not have either focal aware seizures or focal impaired awareness seizures. Subsequently, her ALF improved, and she was able to remember instructions given at work. Although the memories lost because of AA were not recovered, she experienced no further amnestic symptoms due to AA. EEG showed a loss of spikes and a decrease in the voltage of after‐slow activity (Figure S1). She did not desire medication for ADHD.

## DISCUSSION

TEA is a concept proposed in 1998,^1^ and since then, numerous case reports of TEA have been published. However, to the best of our knowledge, there have been no case reports of TEA in patients with ADHD. The combination of a detailed interview, EEG examination, and consideration of TEA enabled us to diagnose the patient with TEA and provide treatment accordingly. It is important to distinguish whether forgetfulness is caused by ADHD or TEA because the treatment approaches for the two conditions are distinct. Because patients with ADHD have a higher prevalence of epilepsy than the general population,^5^ TEA must be considered in patients with ADHD who report amnestic symptoms. However, patients who routinely experience forgetfulness because of their ADHD may not notice an exacerbation of forgetfulness due to TEA. Indeed, our patient was largely unaware of the emergence of new amnestic symptoms due to ALF 2 years earlier and thus did not seek help from a clinic. Therefore, clinicians should consider TEA when patients with ADHD, especially those who are middle‐aged,^6^ complain of forgetfulness.

The diagnostic criteria for TEA include a history of recurrent witnessed episodes of transient amnesia. Our patient did not meet the diagnostic criteria for TEA because the amnestic attacks were not witnessed by others. We identified ALF and AA in her clinical history; therefore, we suspected TEA and subsequently started ASM treatment. We are aware of three cases to date who were diagnosed with TEA but did not have apparent amnestic attacks. Two of the three cases with ALF showed an improvement in ALF symptoms following treatment with ASM,^7^, ^8^, ^9^ and one of the two cases with AA showed an improvement in AA symptoms following treatment with ASM.^7^, ^9^ Ukai et al.^6^ proposed a new clinical entity named “TEA complex syndrome” to enable clinicians to provide treatment without overlooking such a condition. Patients who develop ALF or AA, with or without amnestic attacks, should be considered for ASM treatment in accordance with TEA treatment.

## CONCLUSION

In conclusion, patients with ADHD can have comorbid TEA. It is important to note that patients with ADHD may have difficulty recognizing additional forgetfulness caused by TEA. If patients with ADHD show changes in or exacerbation of forgetfulness, clinicians should conduct an interview to determine the presence of ALF and AA and, if necessary, perform an EEG examination.

## AUTHOR CONTRIBUTIONS

**Takashi Fukao:** Conceptualization; literature search; data curation; visualization; writing—original draft. **Masaki Fujiwara:** Conceptualization; project administration; supervision; writing—review and editing. **Yuto Yamada**, **Shinji Sakamoto**, and **Yosuke Matsumoto:** Conceptualization and writing—review and editing. **Manabu Takaki:** Conceptualization; supervision; writing—review and editing.

## CONFLICT OF INTEREST STATEMENT

Takashi Fukao reports honoraria from Sumitomo Pharma outside of the submitted work. Masaki Fujiwara reports honoraria from Mochida and Eisai outside of the submitted work. Yuto Yamada reports honoraria from Meiji Seika Pharma, Sumitomo Pharma, and Lundbeck. Shinji Sakamoto reports honoraria from Yoshitomiyakuhin, Otsuka, Meiji Seika Pharma, Eisai, Viatris, Tsumura, Sumitomo Pharma, Lundbeck, and Takeda. Yosuke Matsumoto reports honoraria from Eisai, Otsuka, Glaxo Smith Kline, Sumitomo Pharma, Kyowa, Yoshitomiyakuhin, and Viatris. Manabu Takaki is an Editorial Board member of *Psychiatry and Clinical Neurosciences Reports* and a coauthor of this article. To minimize bias, he was excluded from all editorial decision‐making related to the acceptance of this article for publication. He reports honoraria from Otsuka, Sumitomo Pharma, Tsumura, Lundbeck, Merck Sharp & Dohme, Eisai, Meiji Seika Pharma, Viatris, Mitsubishi Tanabe Pharma, Janssen, Yoshitomiyakuhin, and Takeda. He has received unrestricted research funding from Otsuka, Sumitomo Pharma, Eisai, and Mochida.

## ETHICS APPROVAL STATEMENT

N/A

## PATIENT CONSENT STATEMENT

The participant consented to the submission of the case report to the journal.

## CLINICAL TRIAL REGISTRATION

N/A

## Supporting information

Supporting information.

## Data Availability

N/A
