# Supplementary material for: Forgetfulness in adult attention‐deficit/hyperactivity disorder masks transient epileptic amnesia: a case report
Source: PCN Rep. 2024 Aug 22;3(3):e70003. doi: 10.1002/pcn5.70003 (PMC11341431; doi:10.1002/pcn5.70003)

Supplementary Figure 1

Electroencephalography (average reference) showing a loss of spikes and a decrease in the voltage of after-slow activity over the right temporal region (arrows). Yellow vertical lines represent 1-second intervals. Sensitivity = 10 μV/mm; high-frequency filter = 60 Hz; low-frequency filter = 0.53 Hz

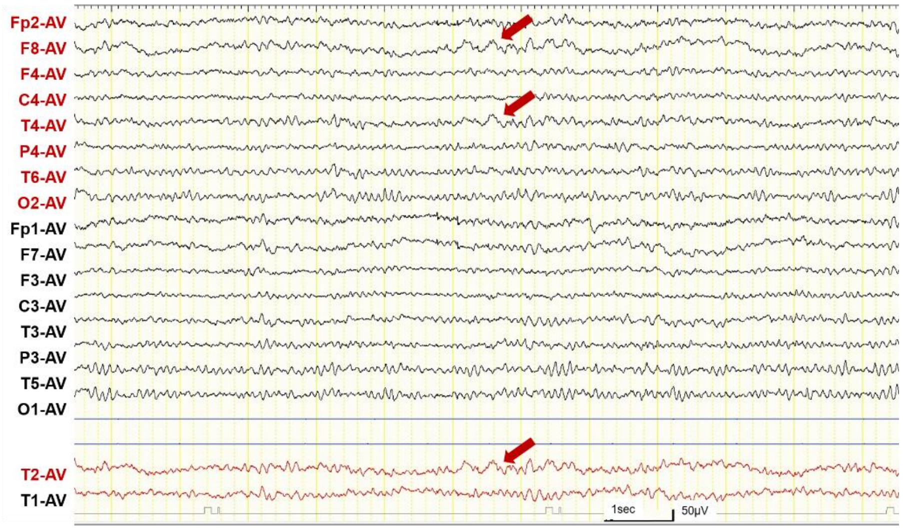

Supplement: Supplementary file 1 — Supporting information. [file PCN5-3-e70003-s001.pdf]
